# The emerging role of dietary fructose in obesity and cognitive decline

**DOI:** 10.1186/1475-2891-12-114

**Published:** 2013-08-08

**Authors:** Shaheen E Lakhan, Annette Kirchgessner

**Affiliations:** 1 Global Neuroscience Initiative Foundation, Los Angeles, CA, USA; 2 Neurological Institute, Cleveland Clinic, 9500 Euclid Ave, S100A, 44195, Cleveland, OH, USA; 3 School of Health and Medical Sciences, Seton Hall University, South Orange, NJ, USA

**Keywords:** Obesity, Nutrition, Cognition, Fructose, Diabetes, Dementia, Omega-3 fatty acids

## Abstract

The incidence of obesity has increased dramatically over the past several years, and in parallel, so has the prevalence of type 2 diabetes (T2D). Numerous studies have demonstrated that both obesity and T2D are associated with lower cognitive performance, cognitive decline, and dementia. Intake of dietary fructose has also increased. In fact, high-fructose corn syrup (HFCS) accounts for as much as 40% of caloric sweeteners used in the United States. Given the increase in the incidence of Alzheimer’s disease (AD), characterized by an age-related decline in memory and cognitive functioning, in this report we review the effects of obesity on cognitive performance and the impact of high fructose intake in promoting cognitive decline. The paper then considers the effects of omega-3 fatty acids (FAs), which have been linked to promising results in cognitive function including ameliorating the impact of a high-fructose diet.

## Background

Obesity is a global health issue that has reached epidemic proportions. Greater than 60% of adults living in the U.S. and Europe are obese (body mass index (BMI) ≥30 kg/m^2^) [1-4]. Moreover, if obesity continues to increase at the present rate, projections indicate that more than 2.3 million adults will be obese by 2015 [5]. Childhood obesity has also become a global health issue and its incidence is increasing in both developed and developing countries. Available estimates (1980–1990) show that the incidence of childhood obesity increased by two to five times in developed countries, and almost four times in developing countries (e.g., from 4% to 14% in Brazil) [6]. An estimated 18% of U.S. children and adolescents are classified as obese [7]. In a recent study, the prevalence of overweight/obesity among American and Hispanic elementary school students in grade 4 was 44.5% [8].

Over the last three decades, the incidence of global diabetes has more than doubled, with nearly 1 in 10 adults affected worldwide [9]. Among U.S. veterans aged 65–85 years, ~30% had diabetes in 2010 [10], of which the overwhelming majority of the cases were type 2 (T2D). The global prevalence of diabetes for all age groups is projected to rise from 171 million in 2000 to 366 million in 2030 [11]. This is not surprising since obesity is a major risk factor for T2D. In fact, the diabetes epidemic appears to have gone hand-in-hand with the increases in obesity. T2D is linked to metabolic syndrome (MetS), defined by a cluster of symptoms including abdominal obesity, insulin resistance, hyperglycemia, dyslipidemia, and hypertension [12], most commonly associated with cardiovascular disease. The incidence of MetS is also rising. According to the Centers for Disease Control and Prevention, approximately 34% of American adults meet the criteria for MetS [13]. Moreover, nearly one third of overweight/obese adolescents had pediatric MetS [14]. Thus, chronic diseases, such as T2D and MetS, once observed solely in adulthood, are now commonly seen among the obese in childhood and are likely to track into adulthood.

Recent studies indicate that both obesity and T2D are associated with cognitive decline. Not only does midlife obesity increase the risk of developing late-life dementia [15,16], but also lower cognitive performance earlier in life is itself a risk factor for dementia later in life [17]. Interestingly, the fastest decline in cognitive function occurs in those with both obesity and metabolic abnormalities; however, individuals who were obese without metabolic abnormalities, still developed a significant cognitive decline [18]. T2D is associated with reduced cognitive function [19] and a increased (1.5-2.5-fold) risk of dementia [20]. Impaired cognitive function is frequently exhibited by individuals with poorly controlled diabetes [21]. Cognitive impairment is also linked to MetS [22], and individuals with MetS in midlife are at increased risk of later life cognitive decline and dementia [23]. Similarly, the incidence of Alzheimer’s Disease (AD), characterized by a progressive and debilitating decline in memory and cognitive functioning beyond that which is normally experience with aging is projected to rise, increasing nearly four-fold over the next 40 years [24]. Thus, identifying the risk factors for delaying the onset and preventing the progression of these disorders is of utmost importance.

Although several factors, such as a lack of exercise, are likely contributors to the rising trend in obesity, increases in its prevalence are directly attributable to excessive caloric intake [25]. In addition, there is now strong evidence that excessive consumption of added sugars (sucrose and high-fructose corn syrup (HFCS)), contributes to rising obesity and diabetes rates [9,26]. There has been a substantial increase in the amount of HFCS found in the North American diet, primarily in soft drinks and fruit drinks [27]. Moreover, findings from large cross-sectional and prospective studies show that increased consumption of sugar-sweetened beverages is positively associated with obesity in both children and adults [28]. Thus, consumption of sugar-sweetened beverages appears to be a key contributor to the epidemic of overweight and obesity. In fact, countries with higher availability of HFCS have a higher prevalence of T2D compared to countries with low availability [29,30]. Since this difference was retained or strengthened after adjusting for BMI, the data suggest that countries with higher availability of HFCS have a higher prevalence of T2D independent of obesity [30]. Abundant consumption of fructose is also an important contributor to the MetS [31]. This raises concerns regarding the short and long-term effects of fructose in humans, and the possibility that fructose intake in childhood is associated with poorer academic performance and impaired cognitive function as an adult.

In this paper we review the association of obesity with cognitive performance. Since high fructose intake appears to be related to the current obesity epidemic, we also review the impact of added sugars on cognitive function and the beneficial effects of dietary supplementation of omega-3 fatty acids (FAs) [32-34].

### Proper nutrition is essential for cognitive function

There is no doubt that proper nutrition is essential for cognitive function. Data suggest that the influence of nutrition on cognitive function begins during fetal life [35,36]. A negative cognitive effect of prenatal caloric restriction has been shown in animals, and studies in humans demonstrate prenatal diet variation in micronutrients negatively affecting childhood cognitive function [37-41]. Prenatal under nutrition also negatively influences cognitive function in later life [36]. de Rooij et al. [36] showed that individuals experiencing famine during the early stage of gestation performed worse on a selective attention task. Since performance on this task usually declines with increasing age, the researchers hypothesized that this decline may be attributed to an increased cognitive aging process [36].

In contrast to the detrimental effects of inadequate nutrition on cognitive function, pre- and postnatal nutritional supplementation enhances cognitive development. In a longitudinal investigation of nutrition and mental development, Freeman et al. [42] found an association between nutritional status and cognitive measures, independent of social factors. Language scores in 3- and 4-year-old children in rural Guatemalan villages were higher in the offspring of mothers who received a high-protein calorie supplement during pregnancy and lactation than in the offspring of mothers who did not. Mothers supplemented with a high calorie diet consumed at least twice as many calories as mothers supplement with a low calorie diet, which may have also contributed to the results.

Even skipping breakfast, which occurs at a rate of 10-30% among children and adolescents in the U.S and Europe [43-45], affects cognitive functioning [46,47]. Skipping breakfast has been shown to negatively affect performance on measures of IQ, the Hagen Central Incidental Test, and the Matching Familiar Figure Test [48]. In contrast, breakfast has beneficial effects on cognitive function. In an internet based study involving children aged 6-16 years, children who ate breakfast performed better on tests of attention and memory than children who skipped breakfast, indicating that breakfast exerts a beneficial effect in maintaining cognitive function during the morning [49]. A very recent cross-sectional study of children aged 6 years showed that children who regularly have breakfast had significantly higher verbal and IQ test scores compared to children who “sometimes” have breakfast [50]. In addition, in a randomized, crossover trial involving high school students (13-20-years-old), breakfast had positive short-term effects on visual-spatial memory and self-reported alertness; however, breakfast had no effect on sustained attention [51].

### Obesity and cognition

There is no doubt that nutrition is critical for cognition and academic performance; however, food intake in excess of energy needs is linked to the development of obesity [52] and obesity has been associated with poorer cognitive function [53,54].

In 2005, Jeong et al. [55] examined the association between obesity and cognition using data from a community study of South Korean adults (≥ 65 years). Cognitive function was examined using the Korean Mini-Mental State Examination, and obesity using BMI and waist circumference, as measured at the level midway between the lower rib margin and the iliac crest [56]. Obesity (BMI ≥ 25 kg/m^2^) and poor cognitive performance were associated with above normal waist circumference, and poor cognitive performance was negatively associated with overweight (BMI, 23-25 kg/m^2^) and normal waist circumference. Recently, Benito-Leon et al. [57] assessed cognitive function in a large population-based sample of overweight (BMI 25-29 kg/m^2^) and obese (BMI ≥ 30 kg/m^2^) elderly (≥ 65 years) participants compared with normal weight controls living in the same community in central Spain (the Neurological Diseases in Central Spain study (NEDICES)). Subjects underwent assessment of cognitive function, which included the 37-item Mini-Mental State Examination (37-MMSE), a test that measures global cognition, tests of psychomotor speed, verbal fluency, memory and pre-morbid intelligence. The study found that obese/overweight status was associated with the lowest quartiles of the 37-MMSE, the Trail Making Test-A, verbal fluency, delayed free recall, and immediate logical memory and pre-morbid intelligence, indicating that overweight and obese participants performed poorer on cognitive tests than their normal weight counterparts. In other studies different aspects of cognition have been found to be negatively associated with BMI including memory [58,59], vocabulary [60], speed processing and reasoning [60]. These results support the association of obesity and impaired cognition in the elderly.

There is also evidence to support the association of obesity with poorer cognitive performance in midlife. A higher BMI in midlife was found to be associated with lower cognitive scores both in cross-sectional [58] and longitudinal [61] analyses. For example, in a prospective cohort study, Cournot et al. [58] analyzed data from healthy, non-demented, middle-aged men and women at baseline (32 to 62 years) and at follow-up, five years later, to assess the association between BMI and cognitive function. After adjustment for co-variables, including age, sex, educational level, blood pressure, diabetes, and other psychosocial factors, a higher BMI at baseline was associated with lower cognitive scores. Moreover, a higher BMI at baseline was associated with lower cognitive scores at follow-up, 5 years later. The association of BMI with decreased cognitive performance was significant for word-list learning (4 recalls). Several other investigators have reported similar results [54,58,60,62]. A higher BMI was associated with lower cognitive function scores in healthy, black and non-black adults (≥ 65 years); however, greater BMI was not predictive of cognitive decline as measured by the MMSE at an average follow-up of 6.4 years [62]. Thus, in this study, greater BMI in old age was not predictive of cognitive decline at follow-up.

There is also data to suggest that obesity is associated with impaired cognitive function in children [63]. Obesity has been linked to poorer academic achievement in school age children as well as greater destruction of brain architecture and function during aging [64]. Decreased amplitude and prolonged latency of P300 auditory event-related potentials were observed in children with obesity compared to that of healthy controls [65]. This difference in recordings of P300 auditory event-related potentials in obese children suggests impairment in cognitive functions [66]. Obese children in kindergarten up until the end of third grade, exhibit lower academic test scores [67]. Additionally, severely obese children have been reported to have lower IQs, display poorer school performance, and lower test scores than their overweight classmates [67]. In addition, adolescents with MetS had significantly lower spelling, attention, arithmetic, and overall mental flexibility with a trend for lower overall intelligence [68]. Furthermore, there is an inverse childhood full IQ/obesity association in adults [63]. However, after adjusting for educational attainment, the full IQ/obesity association was not significantly different [63], suggesting that educational level plays a role in the persistence of obesity in later life.

### Obesity, dementia and cognitive decline

Several studies have shown that obesity in midlife is associated with increased risk of dementia in later life [55,69-85]. A 27-year longitudinal, population based study [81] showed that obese people (BMI ≥ 30 kg/m^2^) had a greater risk of dementia (74%) compared with those who were overweight (BMI 25-29.9 kg/m^2^) or normal body weight at 40-45-years of age (35%) [81]. Less is known about the association between obesity and cognitive aging. “Cognitive aging” refers to the inevitable cognitive decline that occurs with aging, with no evidence of dementia. The extent to which cognitive decline occurs is highly variable, and strongly affected by various disease processes. In general, cognitive abilities remain stable through adulthood and decline around the age of 65 years [86,87].

In the Swedish Adoption/Twin Study of Aging (SATSA) [88], the association between BMI and cognitive decline was examined among participants with an average age of 42. BMI and global cognitive ability were measured over a 20-year period beginning at age 25. The study showed that higher BMI was inversely related to global cognitive ability with longitudinal decline for both women and men as indicated by latent growth curve analyses. Early midlife obesity was associated with a steeper decline in general cognitive ability and perceptual speed, with a tendency for a steeper decline in verbal and spatial abilities. Moreover, being stably obese over a longer period of time was associated with lower cognitive function in late life. In contrast, when obesity developed in late life, there was a steeper decline in only verbal ability. Since verbal ability remains stable over the adult life span until very old age [86], this can be seen as evidence that obesity is associated with cognitive decline. However, most studies assessing obesity in late life found no association between late-life obesity and late-life cognitive abilities [62,74]. Dahl and Hassing [74] conducted a systematic review to examine the association between obesity and cognitive aging among individuals without dementia. The reviewed studies showed clear evidence that midlife obesity was associated with cognitive aging, whereas this association was weaker in late life.

Sabia et al. [53] employing the Whitehall II cohort [18], determined whether cognition in late midlife is influenced by lifetime obesity. In this study, BMI at 25 years (early adulthood) was self-reported at baseline and was measured in early midlife (mean age, 44 years) and again in late midlife (mean age, 61 years). Cognition was evaluated in late midlife by using the 30-item MMSE, and tests of memory (verbal memory) and executive function (Alice Heim 4-I, tests of verbal and phonemic fluency). The study found that the proportion of the population that was either overweight or obese increased from 13.6% (age 25) to 63.1% (age 61). Moreover, long-term obesity in adulthood was associated with lower cognitive performance in late midlife. Those who were obese at 2 or 3 occasions had lower mean MMSE scores and scores of memory and executive function, adjusted for age, sex, and education, than did their normal-weight counterparts. Thus, an increase in BMI over the adult life course was associated with lower cognitive performance in late midlife, supporting earlier reports [58]. These data suggest that the association between obesity and cognition starts early in adulthood and continues to develop for many years.

### Obesity and cognition: the influence of metabolic factors

Singh-Manoux et al. [18], employing the Whitehall II cohort, determined the association of BMI and metabolic status with cognition. A cognitive test battery consisting of 4 standard tasks was administered, which consisted of the Alice Heim 4-1, a test of reasoning, in addition to a recall test to test short-term memory, and two measures of verbal fluency. In addition, a global cognitive score was created based on the 3 tests mentioned above. The results indicated that at baseline, obese individuals (BMI ≥30 kg/m^2^) had lower cognitive scores than normal weight individuals. Moreover, the baseline differences were maintained over the 10-year follow-up. However, cognitive decline on the global score was only faster among individuals with both obesity and metabolic abnormality. It is important to note, that although this trend was evident for all tests, it was statistically significant only for the global cognitive score.

The influence of metabolic abnormalities on the association between obesity and cognition was also observed in the Italian Longitudinal Study on Aging (ILSA) [89], a prospective study, which investigated the relationship of MetS with the incidence of mild cognitive impairment and its progression to dementia. In this study, the presence of MetS, characterized by abdominal obesity, hypertension, and hypertriglyceridemia, in individuals with mild cognitive impairment (MCI) was associated with a significantly higher incidence rate for progression to dementia over 3.5 years of follow-up. The risk in MCI patients with MetS almost doubled compared with those with MCI without metabolic abnormalities.

Hypertension was found to modulate the association between obesity and cognitive functioning in participants of the Framingham Heart study [90]. In this prospective study, BMI and blood pressure status were related to cognitive performance on tests administered 4-6 years later. The lowest levels of cognitive performance were seen for subjects with both hypertension and obesity as compared to those with either hypertension or obesity or neither of these risk factors. Interestingly, adverse effects of obesity and hypertension on cognitive performance were observed only in aging men. A synergistic influence of concomitant obesity and hypertension on cognition was also observed by Wolf et al. [61]. In this study, which employed the Framingham Offspring Study cohort, midlife measures of obesity and of hypertension were each significantly related to poorer cognitive performance on executive function and visual motor skills. Moreover, the relation of hypertension to cognitive performance was significantly modified by obesity. In addition to MetS and hypertension, diabetes has been linked to poorer cognitive performance; however, in this study, diabetes did not modify the effects of obesity on cognition [54], suggesting that the underlying mechanisms may be different.

### Added sugars and cognition

Obesity is directly attributable to excessive caloric intake [25] and there is now strong evidence that intake of added sugars, mainly fructose and sucrose, contributes to the rising obesity and diabetes rates [9,26]. In particular, high-fructose corn syrup (HFCS)-sweetened beverages play a role in the development of obesity, and increased BMI is associated with their increased consumption. Intake of HFCS-sweetened beverages is also linked to metabolic abnormalities characteristic of the MetS. In addition, several studies have shown that intake of added sugars is associated with lower cognitive function.

### Fructose

Fructose, an isomer of glucose, is a natural sugar found in many fruits. In equal amounts, it is sweeter than sucrose (glucose-fructose) and therefore commonly used as a sweetener. The commercial production of HFCS began in 1967. At that time, the fructose content of the syrup was ~15%. After several modifications, the fructose content was increased to 55% (HFCS-55) and it became the sweetener of choice for the soft drink and ice cream industries. Currently, HFCS accounts for 40% of all added caloric sweeteners [25,91]. Approximately 80% of added sugars in soft drinks, baking products, and ice creams consist of HFCS [25,92,93].

The intake of fructose has significantly increased over the past three decades and is derived largely from sucrose (composed of 50% fructose) and HFCS (42%, 55% or 90% fructose). In the US, yearly per capita caloric sweetener consumption rose around 20% since 1970 [94]. Interestingly, this increase coincides with the obesity epidemic. Data on self-reported food intake from the National Health and Nutrition Examination Survey (NHANES) suggest that ~15% of the US population consumes ≥ 25% of energy from added sugars [92]. The largest source of added sugars in the Western diet is sugar-sweetened beverages, which accounts for more than 10% of energy intake [95]. The consumption of sugar-sweetened beverages increased by ~135% between 1977 and 2001 [76] contributing to an increase in the consumption of all caloric sweeteners of ~83 kcal/person/day [77]. It is estimated that nearly 7% of daily caloric consumption in the U.S. is from HFCS [91]. More concerning is the fact that at least 30% of children (1-5 years of age) consumed sugar-sweetened beverages (soft drinks) [96].

The compositional similarity of HFCS to sucrose suggested that it was safe and would be metabolized in a similar manner. Thus, HFCS, like sucrose, was not perceived to pose a significant health risk, with the single exception of promoting dental caries [97,98]. Additionally, fructose does not directly stimulate insulin secretion and has a low glycemic index, which results in smaller increments in plasma glucose levels in healthy individuals and those with T2D [99]. As a result, fructose was accepted as a beneficial dietary component and fructose was recommended as a sweetener to patients with diabetes. However, there are differences in how these sugars are metabolized and utilized in the body. Meals high in fructose have been shown to reduce circulating insulin and leptin levels in women [91]. In contrast, dietary sucrose increases circulating insulin and leptin levels and inhibits eating, which in turn further inhibits food intake [100]. Accordingly, the level of satiety from fructose intake may be less than that of glucose or sucrose and ultimately add to body weight. In support of this idea, increased fructose intake, particularly in the form of HFCS-sweetened beverages, has been implicated in promoting obesity [101,102]. Results from animal studies showed that rats with access (12 hours) to HFCS gained significantly more body weight than rats given equal access to sucrose (10%), even though they consumed the same number of total calories. Moreover, over the long term, rats with access to HFCS gained significantly more body weight than the sucrose-fed group. The HFCS group exhibited an increase in abdominal fat and elevated circulating triglyceride levels [103]. Studies have also shown that high fructose exacerbated weight-gain in rats that are subsequently maintained on a high-fat diet [104]. Thus, excessive consumption of HFCS may contribute to the rising incidence of obesity.

Intervention studies provide a clear view of the relationship between sugar-containing beverages and body weight in humans. In a well-conducted study by Cox et al. [105] in overweight/obese male and female subjects, consumption of fructose (at 25% of energy requirements for 10 weeks), but not glucose, led to significant decreases in resting energy expenditure, thus contributing to the build-up of excess energy substrates. Furthermore, in one of the population segments at high risk of fructose-related obesity, it was demonstrated that a significant reduction in fructose and/or general sugar intake over a short period of time (3 months) in overweight and obese children might reduce the BMI [106].

Despite these data, we do not have direct evidence that links obesity to the consumption of physiological daily intake of fructose (<100 grams) in humans. In addition, added fructose (< 50 g/day) has no deleterious effect on triglyceride levels, glucose control, or insulin resistance. Nevertheless, consumption of sugar-sweetened beverages is associated with excess calorie intake, and an increased risk of T2D through an increase in body weight [107]. The increasing intake of HFCS paralleled the upward trend in the prevalence of T2D observed in the U.S. during the 20th century [25]. Diets high in added-sugars promote visceral adiposity, dyslipidemia, and insulin resistance/glucose intolerance [28,91,108-111]—all components of the MetS [112]. Moreover, in animal studies, HFCS consumption seems to produce some of these changes associated with the MetS even without increasing the body weight [113]. This has led to the recommendation to limit the daily intake of added-sugars to no more than 10% of total energy [114].

### Fructose and cognition

Results from animal studies suggest that fructose intake may be associated with cognitive decline. Stranahan et al. [115] found that rats fed a high-fat, high-glucose diet and given access to HFCS displayed significantly impaired cognitive function, possibly via the development of insulin resistance. Ross et al. [33] reported that a high fructose diet impairs spatial (hippocampal-dependent) memory in male rats. Their study showed that consuming a high fructose (60%) diet for almost 5 months increased the latency to reach the target and decreased time spent in the target quadrant and the number of target approaches in a spatial water maze probe test. High fructose intake did not influence navigational ability. In addition, the rats were able to learn and retain the location of the platform for short periods of time. Only impairments on the retention tests were recorded associated with high fructose intake (given 48 hours after training), which suggests that consumption of such a diet may impair long-term memory storage or retrieval, or both.

Impaired cognitive function by high fructose was also reported by Agrawal and Gomez-Pinilla [32]. Agrawal and Gomez-Pinilla investigated the impact of a diet deficient in omega-3 FAs (n-3 deficient), to represent the “normal” Western diet, and the MetS on cognition and synaptic plasticity, and concomitant changes in the hippocampus, a region of the brain critical for learning and memory. Rats were randomly assigned to four groups: either with dietary omega-3 FA or a omega-3 FA deficient diet, and each of those were given fructose (15%) or no fructose in their drinking water. Dietary omega-3 FA deficiency impaired spatial learning and memory retention in a Barnes maze, which was further enhanced by the high fructose intake. Changes in cognitive function were accompanied by reduced expression of synaptophysin and synapsin I, synaptic plasticity-associated proteins found in the brain. In addition, high-fructose intake led to insulin resistance and hypertriglyceridemia, suggesting that either factor could impact on cognitive performance (see below). The addition of omega-3 FAs to the diet improved memory and ameliorated the memory impairments induced by fructose intake. All parameters of MetS in the brain related to the fructose treatment were also ameliorated by omega-3 FA intake. Together these results suggest that diets enriched with omega-3 FA can prevent the detrimental effects of fructose on cognition under “normal” conditions, and particularly during metabolic stress [32]. Interestingly, long-term omega-3 FA supplementation has been shown to improve cognitive function in experimental animal models of AD [32].

A recent population-based study investigated the association of fructose intake with cognitive function in humans. Ye et al. [116] examined whether routine intakes of total sugars, added sugars (sucrose and HFCS), sugar-sweetened beverages or sweetened solid foods are associated with declining cognitive function in humans. The study included middle-aged and older Puerto Rican adults (n = 737; mean age 56.3), without diabetes. Total sugars were defined as the sum of three free monosaccharides (glucose, fructose, and galactose) and three free disaccharides (sucrose, lactose, and maltose). Cognitive function was measured with a battery of seven-tests: the MMSE, to assess general cognitive function, a sixteen-word list learning task to assess verbal memory, digit span forward and backward, to assess attention and working memory, clock drawing and figure copying, both to assess visual-spatial organization, verbal fluency, to assess the speed at which one provide exemplars to a category, and the Stroop test, the measure cognitive flexibility, response inhibition and processing speed. Among the participants, ~21% of energy intake was obtained from total sugars, and 12% from added sugars. Fruit drinks, soft drinks, dairy desserts, and sweets provided 22.1%, 12.9%, 11.3% and 10.3% of added sugars, respectively. Total sugars, added sugars, sucrose, glucose and added fructose were each significantly inversely associated with low MMSE scores, in contrast to natural fructose (from fruits and vegetables), which had no effect on cognitive function. Greater consumption of total sugars was also associated with lower word list learning score. These findings suggest that increased consumption of added sugars is associated with lower cognitive function in humans. Interestingly, the association between added sugar intake and MMSE was independent of BMI and age.

### Sucrose and cognition

Several studies have been conducted to explore the association of sucrose intake with cognitive function. Sucrose is composed of 50% fructose. Results from animal studies show that diets with higher sucrose content result in memory impairment. Rats exposed to a sucrose solution (32%) in addition to chow were found to have poorer memory in a novel object recognition task, when compared with rats that only received chow, whereas memory was not affected in rats given a high-fat diet [117]. Interestingly, both the sucrose and high-fat groups became obese when given access to the diets and had increased fasting blood glucose levels; however, only the sucrose fed group showed impairment in object recognition. In addition, the time spent exploring the novel object was negatively correlated with fasting blood glucose levels. Thus, sucrose may be affecting cognitive function independent of its effects on body weight, as the two diets had differential effects on learning performance but not on body weight gain. Several animal studies have shown that Western diet-induced learning and memory impairments can precede the development of diet-induced obesity [24,118]. In addition, the authors hypothesized that the impairment in object recognition in sucrose fed rats, is due, to diet-induced alterations in blood glucose [117]. In another study, young rats fed a supplemental sucrose solution (32%) in addition to chow took significantly more time to find a hidden platform in the Morris Water Maze (MWM), a widely-used task for assessing spatial memory, that is impaired in rats with damage confined to the hippocampus (see [119] for review). Moreover, when tested 10 days after the initial training trials, sucrose fed rats displayed deficits in long-term spatial memory [79]. As in the previous study, fasting glucose levels were significantly higher in the sucrose-fed rats supporting the idea that these cognitive deficits arise from metabolic insults.

Postprandial memory performance after consuming a meal with high or a low glycemic index was examined in adults with well-controlled T2D [120]. It was shown that the high glycemic meal led to poorer performance in memory tests when administered 1-2 hours after eating. Similarly, a diet with a higher glycemic index led to poorer memory performance in normal weight undergraduate women [121] and in non-diabetic individuals, including children [122]. In summary, simple rather than complex carbohydrates may impair postprandial memory performance.

### Underlying mechanisms

There are several possible mechanisms underlying the association between fructose intake, obesity, MetS and cognitive function. Animal studies have shown that excessive caloric intake impairs hippocampal synaptic plasticity suggesting that the hippocampus may be particularly sensitive to changes in dietary energy intake [123-125]. The hippocampus is a brain region critical for learning and memory. Thus, diet-induced changes in hippocampal neuronal plasticity may affect memory and cognition.

Inflammation is correlated with cognitive decline [126] and dementia [123,127,128], and various inflammatory markers are increased in obese relative to lean individuals [129]. Sugar-sweetened beverages were found in a 10-week intervention study to increase inflammatory activity [130]. IL-1 has been implicated in memory consolidation, whereas IL-6 may mediate hippocampal dysfunction and thus affect memory and cognition [131]. IL-6 and C-reactive protein (CRP) correlated with accelerated functional decline in the elderly, and middle age CRP levels may prognosticate dementia risk [132]. Patients with postsurgical neurocognitive decline, which occurs in 7-26% of patients undergoing surgery [133], were observed to have a significant increase in CRP and IL-1 compared to patients without cognitive decline. Postoperative elevations in IL-6 and CRP were found to correlate with short- and medium-term cognitive dysfunction after coronary artery bypass surgery [134].

Vascular pathologies have been hypothesized to play a role [71]. In fact, there is evidence that the relations between obesity and cognition are modified by the presence of cardiovascular risk factors. In the Framingham Heart Study [90], obesity and hypertension were independently associated with lower scores on multiple measures of cognitive functioning, and the adverse effects of obesity and hypertension were additive with respect to measures of episodic memory and visual-spatial constructive abilities. The highest level of performance was observed in the absence of obesity and hypertension, the second highest for those with either hypertension or obesity, and the lowest for those with both obesity and hypertension [90].

It is now known that many of the diagnostic characteristics that define the MetS are individually related to cognitive impairment. Recently, Yau et al. [68] documented lower cognitive performance and alterations in brain structure among adolescents with MetS. Adolescents with MetS scored significantly lower on tests of arithmetic, spelling, attention, and mental flexibility and displayed a trend for lower overall intelligence. They also had, in a metabolic syndrome-dose-related fashion, smaller hippocampal volumes. Excessive fructose intake has been linked to increased lipogenesis, glycogenesis, oxidative stress and uric acid production [135], and these metabolic changes are also associated with the MetS, which in turn, may lead to cognitive dysfunction [136]. Two epidemiological studies have reported an association between elevated uric acid and cognitive decline in older individuals [137,138], whereas one study found a protective effect [139].

Results suggest that insulin resistance induced by high fructose is linked to cognitive decline [140]. Rats fed diets supplemented with HFCS were more insulin resistant and cognitively impaired on spatial learning and ability tasks than non-supplemented rats [115]. Agrawal and Gomez-Pinilla [32] found that fructose intake and DHA deficiency increased hippocampal insulin resistance, as shown by a decrease in insulin receptor signaling. Insulin resistance results from impaired signaling at the level of the insulin receptor primarily as a result of altered phosphorylation. Phosphorylation of the insulin receptor and its distal signaling molecule Akt were reduced in rats fed the omega-3 FA-deficient diet, and that these effects were aggravated by high fructose intake. Inclusion of omega-3 FAs in the diet restored insulin signaling. A role of insulin resistance in memory impairment is supported by the observation that exogenous insulin enhances memory even in individuals with AD [141]. Since memory deficits are positively correlated with increases in insulin resistance, this suggests that insulin signals neurons directly in the brain. Insulin can cross the blood–brain barrier (BBB) [142]. However, insulin receptor knockout mice perform normally in spatial learning and memory tests [143], which suggests that other mechanisms are involved.

In rats, high fructose intake resulted in hyperinsulinemia, hyperglycemia, and an increase in triglyceride (TG) levels [32]. In the study by Agrawal and Gomez-Pinilla [32], insulin resistance index increased in proportion to TG levels, and given the association of cognitive impairment with elevated TG levels, high fructose intake may prime the brain to insulin resistance by its effects on TGs. In fact, the application of TGs to hepatic cells decreases insulin’s ability to trigger its signaling cascade [144]. In addition, TGs can penetrate the BBB [145] and an injection of TGs directly into the brain impairs memory [124]. Data also suggest that neuronal cells can metabolize fructose [146]; however, it is not known whether fructose can penetrate the BBB.

### The beneficial effects of omega-3 FAs

Omega-3 FAs (eicosapentaenoic acid (EPA) and docosahexaenoic acid (DHA)) are diet-dependent factors critical to normal brain development and function [147], that have been shown to exert beneficial effects on cognition. DHA, the principle omega-3 (PUFA) in brain, has effects on neuronal membrane activity, which modulates cell signaling [148]. DHA status is dependent on dietary supplementation because mammals are inefficient at producing DHA from precursors. In humans [149] and rats [150], DHA concentration in the brain decreases with age. This appears to be related to the age-related deterioration in CNS functions [149]. Data from animal studies support this idea. Rodents fed a low omega-3 FA diet showed significant cognitive deficits [150] that were reduced by DHA supplementation [151]. In addition, DHA supplementation improved memory performance in aged mice [152]. DHA levels in the hippocampus decrease with age and AD and impairs hippocampal-dependent spatial learning memory ability. This implies that adequate levels of DHA are required for optimal cognitive performance.

Supplementation with omega-3 FAs can counteract the effects of a high fructose diet on memory [32]. In rodents, dietary omega-3 FA deficiency was associated with a decline in spatial memory in proportion to the intensity of insulin resistance, which was further aggravated by high fructose intake. There was no difference in body weight or total caloric intake; therefore, obesity did not appear to be a major contributor to altered memory functions in this model. Based on these data, it appears that omega-3 FA deficiency increases the vulnerability to the effects of high fructose, as evidenced by disruptions of insulin signaling and impaired cognitive functions.

A potential mechanism has been proposed [153] by which DHA supports the development and maintenance of spatial learning memory. DHA is synthesized and taken up by neurons of the developing brain and hippocampus, which incorporate it into membrane phospholipids, especially phosphatidylethanolamine. This results in enhanced neurite outgrowth, synaptogenesis and neurogenesis. In support of this idea, exposure to omega-3 FAs increased synaptic protein expression to increase the dendritic spine density, and enhanced synaptic plasticity by increasing neurogenesis in the hippocampus. Retinoid signaling may be involved in the effects of DHA on learning memory performance as DHA supplementation appeared to increase retinoid X receptor expression compared with the untreated old group [154].

Dietary DHA appears to be a promising target to protect against the detrimental effects of high fructose intake on cognitive performance. Animal studies have shown that consumption of a DHA enriched diet restores brain DHA levels [155], enhances learning and memory tasks in aged animals [155,156], and significantly reduces beta amyloid, plaques, and tau in transgenic AD models [157,158]. Evidence from clinical studies indicates that dietary supplementation with omega-3 FAs improves cognitive performance in healthy children and adults [159] and is somewhat effective in preventing or ameliorating cognitive decline in the aged [160]; however, these findings are not consistent across studies [161,162]. Dietary supplementation with omega-3 FAs has little or no effects in underperforming individuals, and patients with mild to moderate AD [163]. Nevertheless, individuals (aged 65–94 years) who consumed fish at least once per week were found to have a reduced risk of AD at ~ 4 years follow-up compared to those who rarely or never ate fish [164]. Clearly, additional studies examining the effects of longer duration dietary supplementation with omega-3 FAs may identify greater change in cognitive function in study subjects.

## Conclusion

The association of obesity with chronic diseases, such as T2D and MetS is well known. What has recently emerged is the association of obesity with cognitive decline and that intake of added sugars may mediate the influence of obesity on cognitive function. The intake of fructose, along with HFCS has increased over the past three decades. With the rising trend in childhood obesity, causing children to be at risk of diabetes and MetS, the potential contribution of fructose to lower academic performance in adolescents is becoming increasingly realized. Although obesity may not be enough to warrant concern among parents, the lower academic potential of obese adolescents strongly argues for early treatment of childhood obesity and comprehensive intervention, including a limitation of sweetened soft drinks, especially those containing HFCS. Equally important is exploring the role of dietary omega-3 FAs, which appear to have beneficial effects on cognitive function and attenuate high-fructose associated cognitive decline.

## Abbreviations

AD: Alzheimer’s disease; BMI: Body mass index; CRP: C-reactive protein; DHA: Docosahexaenoic acid; EPA: Eicosapentaenoic acid; HFCS: High-fructose corn syrup; Il-1: Interleukin-1; Il-6: Interleukin-6; ILSA: Italian Longitudinal Study on Aging; Omega-3 FA: Omega-3 fatty acids; SATSA: Swedish Adoption/Twin Study of Aging; T2D: Type 2 diabetes; TG: Triglycerides; MetS: Metabolic syndrome; MMSE: Mini-Mental State Examination.

## Competing interests

The authors declare that they have no competing interests.

## Authors’ contributions

Both authors participated in the preparation of the manuscript, and read and approved the final manuscript.
